# Oncolytic virus therapy in the elderly: immune frailty, challenges, and perspectives

**DOI:** 10.3389/fimmu.2025.1686659

**Published:** 2025-10-08

**Authors:** Jia-Wen Wang, Jia-Hui Liu, Yue-Lin Liu, Wen-Zheng Xu, Zi-Bo Zhang

**Affiliations:** ^1^ Department of Orthopedics, The Fourth Hospital of Hebei Medical University, Shijiazhuang, Hebei, China; ^2^ The Second Hospital of Hebei Medical University, Shijiazhuang, Hebei, China; ^3^ Hebei Medical University, Shijiazhuang, Hebei, China

**Keywords:** immune frailty, elderly cancer patients, oncolytic virus therapy, immunosenescence, inflammaging, cytokine release syndrome

## Abstract

With global aging accelerating, cancer incidence among older adults is rapidly increasing. Individuals aged ≥65 years now represent 64% of new cancer cases and 71.3% of cancer-related deaths worldwide. This population exhibits a distinct immune imbalance—driven by tumor-induced immunosuppression, immunosenescence, and inflammaging—which contributes to poor tolerance of standard therapies and suboptimal outcomes with PD-1/PD-L1 inhibitors.

As an emerging immunotherapeutic strategy, oncolytic viruses (OVs) selectively infect tumor cells, induce immunogenic cell death (ICD), and activate the cGAS–STING pathway. Although clinical data in elderly patients with esophageal, lung, or pancreatic cancer are scarce, promising outcomes have been reported in melanoma/sarcoma subgroups, including objective response rates of 26.4–32.9% and a median duration of response of 33.7 months, highlighting the potent antitumor potential of OVs.

However, age-related immunological vulnerability—manifesting across different frailty stages as reflected by G8 scoring—may predispose elderly patients to immune overload, cytokine storm, and impaired tolerance, while this group remains underrepresented in OV trials. Systematic studies in this context are lacking. This review highlights the immunological characteristics of aging, emphasizes the importance of addressing immunological vulnerability across different age stages (G8 scoring), and outlines emerging challenges and future directions for OV-based therapies tailored to frail elderly populations.

## 1 Introduction

As global aging progresses, the incidence of newly diagnosed cancers is steadily rising. By 2050, it is estimated that approximately 35 million new cancer cases will occur annually worldwide (1). Presently, the elderly population (≥65 years) accounts for about 64% of new cancer cases and 71.3% of cancer-related deaths (2), with these proportions projected to increase further. Elderly cancer patients experience a distinct immune imbalance shaped by tumor-induced immunosuppression, age-associated immunosenescence, and inflammaging (3, 4). This unique immunological state contributes to the high toxicity of conventional therapies, with grade 3–5 adverse events occurring in 53–83% of cases and a treatment-related mortality rate of 2% (5), alongside overall poor tolerance to therapy (6). Moreover, immune checkpoint blockade with PD-1/PD-L1 inhibitors exhibits limited effectiveness in older patients (7) and a higher risk of immune-related adverse events affecting the skin, kidneys, and gastrointestinal tract (8, 9).

Oncolytic viruses (OVs), as a novel class of cancer immunotherapies, selectively infect tumor cells and induce immunogenic cell death (ICD), promoting the release of damage-associated molecular patterns (DAMPs) and tumor-associated antigens. Additionally, they activate the cGAS–STING innate immune pathway and stimulate type I interferon production, thereby converting immunologically “cold” tumors into “hot” ones (10). Notably, in the context of melanoma/sarcoma subgroups, OVs have achieved objective response rates of 26.4–32.9%, complete responses in 15.0%, durable response rates (DRR ≥ 6 months) in 16.3%, and extended the median duration of response to 33.7 months (11, 12). However, the overall incidence of adverse events (AEs) related to OV therapy is 26.6%, nearly 2.07 times higher than in control groups (13). Furthermore, elderly patients remain severely underrepresented in OV trials, especially with those aged ≥70 years comprising only 17.7% of early-phase clinical studies (14). This lack of age-specific data casts doubt on the generalizability of OV findings to older populations.

Due to their distinctive immunosuppressive profiles (15–17), elderly patients undergoing OV therapy may be vulnerable to multiple complications, including the dual burden of immune overload and exhaustion (18, 19), cytokine storm-induced inflammation (18, 20), and compromised immune tolerance (21–23). This complex and multifactorial immune state heightens both the risks and limitations of OV-based therapy in the elderly, posing significant safety and efficacy challenges (24–26). Despite this, there is still a dearth of systematic investigations into the mechanisms of OV therapy under the backdrop of immune frailty in aging populations (14). Most existing studies rely on young or adult models and cohorts, leaving gaps in our knowledge regarding elderly-specific immune microenvironmental changes, virus–host interaction patterns, and optimal treatment timing (27).

**Figure 1:**
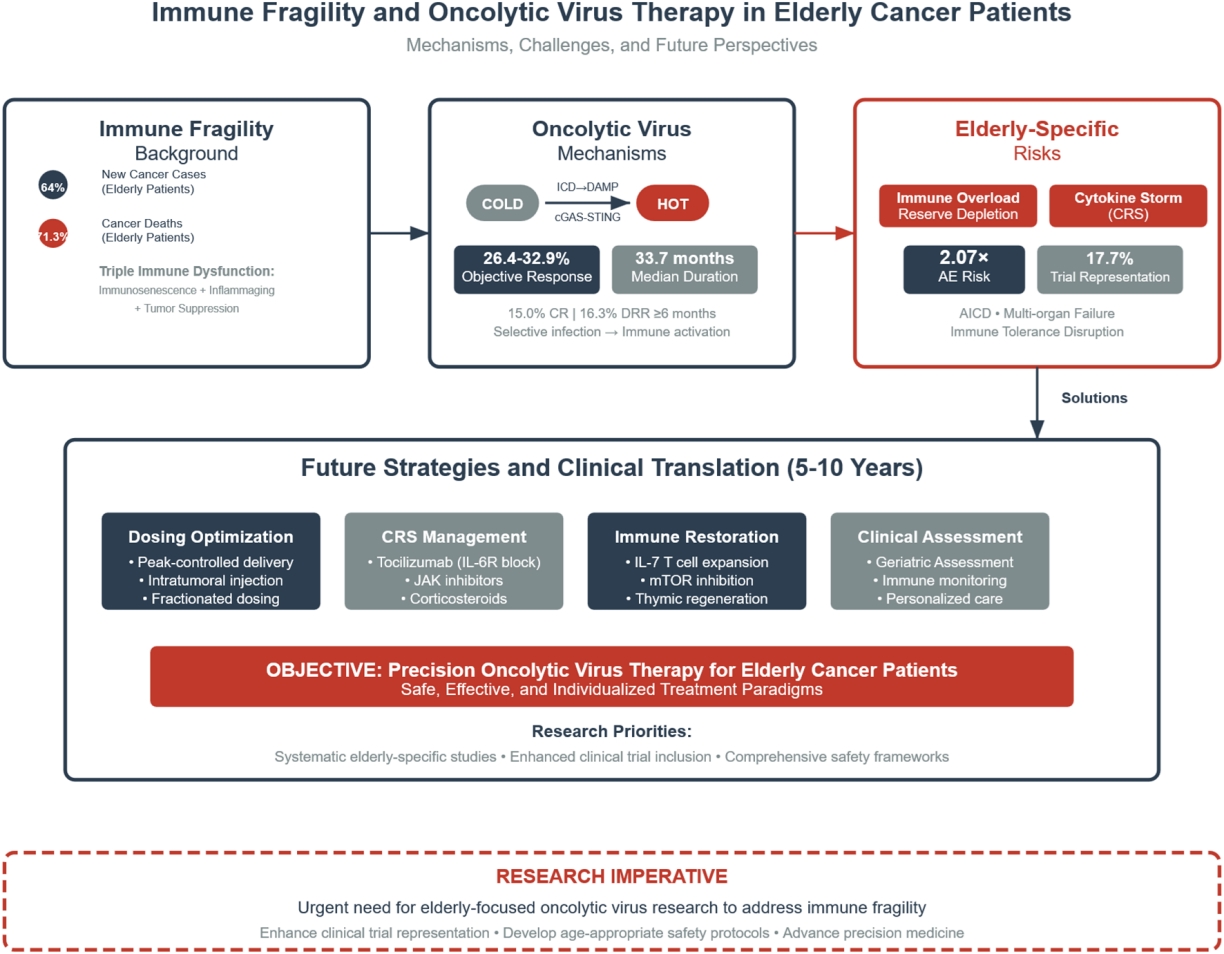
Integrated framework of immune fragility and oncolytic virus (OV) therapy in elderly cancer patients. ICD, immunogenic cell death; DAMP, damage-associated molecular patterns; cGAS–STING, cyclic GMP–AMP synthase–stimulator of interferon genes pathway; CRS, cytokine release syndrome. This figure illustrates the integrated framework of immunological frailty and oncolytic virus (OV) therapy in elderly cancer patients. Elderly individuals account for 64% of newly diagnosed cancers and 71.3% of cancer-related deaths, and exhibit a triple immune imbalance characterized by immunosenescence, inflammaging, and tumor-induced immunosuppression. OVs promote the conversion of “cold” tumors into “hot” tumors via immunogenic cell death (ICD), the release of damage-associated molecular patterns (DAMPs), and activation of the cGAS–STING pathway. Although clinical data remain limited for elderly patients with esophageal, lung, or pancreatic cancers, an objective response rate (ORR) of 26.4–32.9% and a median duration of response (DOR) of 33.7 months have been achieved in melanoma/sarcoma subgroups. However, elderly patients face unique risks, including immune overload and reserve exhaustion, cytokine storm (CRS), and disruption of immune tolerance, with a 2.07-fold increased risk of adverse events and underrepresentation in clinical trials (17.7%). Future strategies should focus on four key areas: optimized drug delivery, CRS management, immune reconstruction, and personalized frailty-based assessment. Dark blue elements indicate core mechanisms, grey indicates neutral or observational data, and red highlights clinical risk warnings. Arrows denote causal relationships and directional processes.

This review seeks to elucidate the immunological features unique to elderly individuals and their interactions with OV therapy, focusing on three key questions (**Figure 1**):

1. What are the pathophysiological characteristics of the immune microenvironment in elderly cancer patients? How do tumor-induced immunosuppression and age-related immunosenescence synergize at the molecular level to create a state of immune frailty? How does immunological vulnerability across different age stages affect immune function?
2. What unique immunotoxicities are associated with OV therapy in elderly patients? What are the mechanisms and clinical manifestations of the vicious cycle of immune overload and exhaustion, cytokine release syndrome, and loss of immune tolerance?
3. Why is it essential to prioritize the unique immune status of elderly cancer patients? Can precision interventions targeting immune frailty improve therapeutic windows? Can integrated strategies—such as immune reconstruction technologies, pharmacological optimization, and stratified management systems—maximize the benefits while minimizing the risks of OV therapy in older adults?How should this be achieved?

### 1.1 Unique immune frailty in elderly cancer patients

The immune microenvironment of elderly cancer patients (≥65 years) is distinctively complex (17), characterized by tumor-induced immunosuppression, age-related immunosenescence, and their synergistic disruption of immune homeostasis. Together, these factors constitute a unique state of immune frailty in elderly cancer patients.

Tumor-induced immunosuppression is commonly observed in this population (**Table 1**). Specifically, cancer cells suppress effector T cell activity and function through the secretion of immunosuppressive cytokines such as transforming growth factor-β (TGF-β), interleukin-10 (IL-10), and vascular endothelial growth factor (VEGF) (28, 29). In addition, they activate suppressive metabolic pathways, including the denosine axis (CD39/CD73 → adenosine) and the tryptophan–kynurenine–AhR pathway (IDO1/IDO2/TDO2 → Kyn → AhR), further impairing T cell function (16). Tumor cells may also directly engage inhibitory immune checkpoints, such as programmed death-ligand 1 (PD-L1) and cytotoxic T lymphocyte-associated protein 4 (CTLA-4), thereby inducing T cell apoptosis (30). Concurrently, the tumor immune microenvironment recruits and activates suppressive cell populations such as regulatory T cells (Tregs) and myeloid-derived suppressor cells (MDSCs), both of which significantly inhibit antitumor immunity (31). The situation is further compounded by pro-inflammatory cytokines secreted via the senescence-associated secretory phenotype (SASP), including IL-6, IL-8, and TGF-β, which synergize with tumor-derived signals to exacerbate MDSC and Treg-mediated immunosuppression (17). This immunosuppressive milieu is frequently observed in elderly cancer patients and is associated with a reduced 5-year survival rate of just 38–50% among breast cancer patients aged over 70 years (32).

**Table 1:** Tumor-induced immunosuppression in elderly cancer patients.

Mechanism	Specific details	Evidence	Ref.
Co-upregulation of immune checkpoints and tryptophan metabolism → suppressive microenvironment	PD-L1↑, IDO1↑	Integrated immunogenomic analysis indicates that with aging, PD-L1 and IDO1 expression increases in normal brain tissues, accompanied by elevated peripheral Tregs and reduced cytotoxic CD8^+^ T cells, especially in the 60–69 age group. These findings suggest a link between aging (≥65 years) and enhanced immunosuppression, consistent with the peak incidence of glioblastoma.	(15, 111)
Treg-driven immunosuppression within tumors	Treg accumulation/activation	In a cohort of ≥70-year-old breast cancer patients (n=40), individuals lacking Her2-reactive CD8^+^ T cells and exhibiting high Treg levels had a 5-year survival rate of 50%, compared to 100% in those with both Her2 responsiveness and low Tregs (P=0.03). This implies Treg-associated suppression correlates with worse outcomes.	(32)
Tumor-induced myeloid suppression	MDSC expansion	The same elderly breast cancer study found that patients with absent Her2-reactive CD8^+^ T cells and elevated Lin^-^CD14^+^HLA-DR^-^ MDSCs had a 5-year survival of 38% versus 100% (P=0.03), highlighting the clinical relevance of MDSCs in older patients.	(32)
Adenosine-mediated immunosuppressive axis	CD39/CD73 → adenosine (A2AR)	A stratified study in head and neck squamous cell carcinoma (HNSCC) (young vs. ≥70 years) revealed significantly higher Treg infiltration in tumors than in peripheral blood. Aging was associated with elevated PD-1 on peripheral T cells and decreased CD73 expression. Mechanistic reviews confirm CD39/CD73/A2AR signaling contributes to immunosuppression across multiple cancers and represents a clinical target.	(16, 112)
Kynurenine–AhR pathway	IDO1/IDO2/TDO2 → kynurenine → AhR	Histological analysis in glioblastoma (n=108) revealed high IDO1/IDO2/TDO2 and AhR expression correlated with significantly worse overall survival (multivariate: IDO1 HR 3.39; IDO2 HR 2.78; AhR HR 1.90). Dysregulated kynurenine pathway (KP) is a key driver of tumor immune evasion and is linked to advanced age.	(113)
Senescent microenvironment amplifies tumor-induced suppression	SASP cytokines (e.g., IL-6, IL-8, TGF-β) → MDSC/Treg recruitment and suppressive cascades	Reviews of aged tumor microenvironments show that senescence-associated secretory phenotype (SASP) factors synergize with tumors to enhance MDSC/Treg-mediated immunosuppression, thereby impairing antitumor immunity and immunotherapy responses. This is a key component of "immune frailty" in elderly patients.	(17)
Checkpoint exhaustion (age-enhanced)	PD-1 overexpression / contraction of effector CD8^+^ T cells	In elderly HNSCC patients, PD-1 levels on peripheral T cells were higher than in younger individuals, and PD-1 expression was even more pronounced on tumor-infiltrating T cells. This suggests tumor-induced exhaustion is exacerbated in older adults and theoretically indicates checkpoint inhibitor responsiveness.	(16)

Age-related immunosenescence leads to thymic involution and a marked reduction in naïve T cells and T cell receptor (TCR) diversity and quantity, thereby compromising antigen recognition and immune responsiveness (15, 33). Metabolic reprogramming also occurs in aged T cells, affecting mitochondrial function, glycolytic pathways, and chromatin accessibility, which diminishes the quality of both effector and memory T cells (15, 34). In addition to T cell defects, age-related B cells (ABCs), particularly the T-bet+CD11c+ phenotype, become more prevalent, weakening humoral immune responses and impairing antigen-specific and antitumor immunity (35). Hematopoietic stem cells (HSCs) in the elderly exhibit a “myeloid bias” (19), resulting in increased MDSC production and activity, suppression of T cell function, and enhanced Treg expansion (36). Aging also impairs dendritic cell (DC) maturation, chemotactic ability, and cytokine secretion, leading to both quantitative and functional decline. Although natural killer (NK) cell numbers may increase with age, their cytotoxicity and cytokine production capacity are significantly diminished, compromising the first line of defense against tumors (37, 38). Moreover, elderly individuals often present with “inflammaging,” a pro-inflammatory state strongly associated with adverse outcomes such as frailty and mortality. This state is marked by chronically elevated levels of IL-6 and TNF-α (39), which further promote myeloid skewing and MDSC expansion, suppress adaptive immunity, and contribute to a tumor microenvironment characterized by both immunosuppression and chronic inflammation (40). These immunosenescence-related changes collectively result in profound immunosuppression (**Table 2**).

**Table 2:** Age-related immune system decline in elderly cancer patients.

Mechanism	Specific details	Evidence	Impact	Ref.
Thymic involution → reduced naïve T cells and TCR diversity	Reduced thymic output, fewer RTEs/naïve T cells, narrowed TCR repertoire	Consistent findings across studies indicate that aging leads to significant reductions in thymic output, peripheral naïve T cells, and TCR diversity	Impaired recognition of novel antigens (including tumor neoantigens), weakened primary responses, and compromised antitumor immunity initiation	(15, 33, 114)
T-cell subset remodeling and accumulation of senescent phenotypes	Decline in CD8^+^ naïve T cells; increase in CD8^+^CD28^-^/CD57^+^/KLRG1^+^ senescent/terminal memory-like cells; rise in PD-1^+^ T cells	Metabolic and transcriptional analyses show aged naïve T cells exhibit impaired TCR signaling and mitochondrial biogenesis	Limited effector T-cell priming, expansion, and memory formation; response to ICB therapy becomes more context-dependent	(15)
Myeloid-biased hematopoiesis	Increased HSC frequency with a bias toward myeloid differentiation	A 2011 PNAS study demonstrated a significant increase in “myeloid-biased” HSCs with age	Promotes accumulation of tumor-associated suppressive myeloid cells and contributes to a fragile tumor microenvironment (TME) characterized by both inflammation and immunosuppression	(19)
Expansion of myeloid-derived suppressor cells (MDSCs) with age	Increased MDSC numbers and function in peripheral and secondary lymphoid tissues	Multiple reviews and empirical studies suggest aging and chronic low-grade inflammation drive MDSC expansion	MDSCs suppress T-cell activity, promote Treg expansion, and facilitate immune escape, weakening antitumor immunity and impairing vaccine/immunotherapy efficacy	(36, 115)
Decrease in dendritic cell (DC) quantity/function	Reduced maturation markers, chemotactic ability, and effector cytokine production	Foundational reviews show that aging and cancer both impair DC quantity and quality	Compromised antigen presentation and naïve T-cell priming hinder neoantigen-driven antitumor responses	(116)
Functional decline in NK cells	NK cell numbers may increase, but cytotoxicity and cytokine production decline	Systematic impairment of NK phenotype and function with age has been observed	Reduced immune surveillance against MHC-I-deficient tumor cells facilitates immune evasion	(37, 117)
B-cell aging and accumulation of age-associated B cells (ABCs)	Significant rise in ABCs (e.g., T-bet^+^CD11c^+^) with aging; decline in humoral immunity quality	ABCs are elevated in aged and disease populations; correlations also observed in cancer immunotherapy cohorts	Impairs antigen-specific humoral immunity and vaccine response quality; inflammatory/autoimmune-like signals may disrupt antitumor immunity	(35, 118)
Inflammaging	Chronic elevation of IL-6, TNF-α, and other proinflammatory cytokines	Large cohort studies link elevated IL-6 to adverse outcomes in elderly individuals	Promotes myeloid skewing and MDSC expansion; inhibits adaptive immunity; shapes an “inflammatory-suppressive” TME	(39)
Metabolic and epigenetic immune senescence	Alterations in mitochondrial function, glycolysis/PPP, and chromatin accessibility	Integrative reviews link age-associated metabolic changes to T-cell function decline	Restricts effector and memory T-cell quality; impairs sustained control over tumor antigens	(15, 34)
Global decline in innate immune function	Multi-dimensional changes in numbers, receptors, and effector functions of neutrophils, macrophages, DCs, NKs	Human studies summarize the pervasive impact of aging on innate immunity	Disruption of innate–adaptive immune crosstalk; impairs early tumor recognition and activation of adaptive immunity	(117)

When tumor-induced immunosuppression coexists with age-related immunosenescence, their interplay gives rise to a vicious cycle of mutual reinforcement (**Table 3**). Chronically elevated IL-6 activates the JAK/STAT3 signaling pathway, upregulating PD-L1 expression on tumor cells. It also inhibits tumor antigen presentation via the NMD/SMG1 pathway, thereby facilitating immune escape and further weakening antitumor immunity (41, 42). SASP exacerbates immunosuppression by increasing the number of MDSCs and Tregs (17), upregulating Treg expression and FoxP3 levels (43). Tumor-derived extracellular vesicles (tEVs) carrying PD-L1 can induce T cell DNA damage and lipid metabolism reprogramming, thereby accelerating T cell senescence (44). Simultaneously, aged microenvironments release extracellular vesicles (aged-EVs) that remodel the tumor milieu to favor cancer progression. When combined with tEV-PD-L1, these factors further intensify immunosuppression (45). Tumor-secreted suppressive factors such as VEGF aggravate the existing decline in antigen presentation and IFN production capacity (46), leading to severely compromised tumor antigen recognition and presentation by antigen-presenting cells (APCs) like DCs (47).

**Table 3:** Synergistic effects between tumor-induced immunosuppression and immunosenescence in elderly cancer patients.

Mechanism	Specific details	Evidence	Study subjects	Ref.
Inflammaging → Tumor Immune Evasion	IL-6/JAK/STAT3 axis upregulates PD-L1 and suppresses anti-tumor immunity; STAT3 also inhibits neoantigen presentation via NMD/SMG1 pathway	IL-6 dose-dependently activates STAT3 and increases PD-L1 expression (in vitro); IL-6/STAT3–induced SMG1 limits frameshift neoantigen expression, weakening immune responses (human cohorts & mechanistic studies)	Osteosarcoma, gastric cancer	(38, 41, 42, 119)
SASP → Expansion of Immunosuppressive Cells	SASP factors (IL-6/IL-8/IL-10) in aged TME recruit and expand MDSCs/Tregs while suppressing effector T/NK cytotoxicity	Aged tumor-bearing mice show significant MDSC accumulation in bone marrow/blood/spleen; Tregs and FoxP3 levels are elevated in elderly lung cancer patients and Lewis lung carcinoma models	Multiple cancers; Lewis lung cancer; human cohorts	(43)
Tumor-derived EVs (tEVs) → T Cell Senescence/Exhaustion	tEV-PD-L1 induces T cell DNA damage and lipid metabolic reprogramming → senescence; inhibition of EV synthesis or lipid metabolism restores T cell function and enhances anti–PD-L1 efficacy	In both human/mouse melanoma models, tEV–PD-L1 drives T cell senescence; combining metabolic or EV inhibition improves immunotherapy response	Melanoma (human & mouse)	(44, 120)
Tumor-secreted VEGF × Age-related DC Dysfunction	Tumor-derived VEGF impairs DC maturation; aging further reduces DC antigen presentation and IFN production → synergistic inhibition of T cell priming	Classical studies confirm VEGF-induced DC dysfunction; aged human/mouse DCs show reduced antigen presentation and cytokine secretion	Multiple cancers	(46, 121, 122)
Aged Treg Accumulation × Tumor-induced Tolerance	Increased Tregs in aged spleen/lymph nodes/tumors reinforce tumor-induced tolerance and suppress effector T cells	Elevated Treg infiltration and FoxP3 mRNA in elderly Lewis lung cancer models and patients; multiple studies show age-related Treg expansion	Lung cancer (human & mouse)	(43)
Aged Fibroblasts/Matrix Remodeling × Tumor Immunosuppression	Aged fibroblasts secrete sFRP2 and lipids, reshaping ECM/metabolism, promoting metastasis/drug resistance and suppressing immunity	sFRP2 elevation promotes melanoma metastasis and resistance; aged TME alters immune landscape and therapeutic efficacy	Melanoma	(123, 124)
T Cell Repertoire Attrition × Tumor PD-1/PD-L1 Axis	Thymic involution and naïve T cell output decline, with accumulation of exhausted phenotypes (PD-1↑, KLRG1↑), making them more susceptible to PD-L1–mediated suppression	Reviews and multiple human/mouse studies show CD27/CD28 loss and PD-1 upregulation in aged T cells; thymic atrophy reduces TCR diversity	Multiple cancers	(43)
Aging × Efficacy of Immune Checkpoint Therapy	Aging alters Treg/CD8 ratios and IFN signaling, leading to heterogeneous PD-1/PD-L1 responses	In melanoma, patients >60 years respond better to anti–PD-1 due to reduced Treg:CD8 ratios (human & mouse, n=538); aged TNBC mice show poor response to anti–PD-L1	Melanoma, TNBC	(43, 125)
Aged Extracellular Vesicles (aged-EVs) × Tumor-permissive TME	Aged tissue–derived EVs remodel the TME to promote tumor permissiveness, synergizing with tEV-PD-L1 effects	Aged-EVs create a tumor-permissive microenvironment (in vitro & in vivo)	Multiple cancers	(45)

Moreover, the degree of frailty across different stages of aging significantly impacts the immune system of elderly cancer patients (**Table 4**). Patients with mild frailty (65–74 years, G8 score 15–17) typically exhibit only mild thymic involution. Naïve T cells are relatively preserved, baseline inflammatory markers such as IL-6 remain low (<5 pg/mL), MDSCs show only slight increases, and Tregs expand modestly (baseline +10–20%). As a result, these patients retain a 25–30% response rate to immunotherapy (48, 49). In contrast, moderately frail patients (70–84 years or ≥3 comorbidities, G8 score 11–14) exhibit more pronounced thymic atrophy, significant reductions in TCR diversity, elevated inflammatory load (IL-6 at 5–15 pg/mL), 1.5–2-fold expansion of MDSCs, and a 30–50% increase in Tregs, resulting in reduced immunotherapy response rates of 15–25% (43, 50). Severely frail patients (≥85 years or G8 score ≤10) show marked thymic atrophy, accumulation of terminally differentiated T cells (notably increased CD28–CD57+), and severe baseline inflammation (IL-6 >15 pg/mL). MDSCs are elevated by more than 2-fold, and Tregs expand by 50–80%, leading to immunotherapy response rates dropping below 15% (34, 51). These frailty-related immune differences across age groups directly affect the efficacy of OV therapy by modulating immune responsiveness, ultimately influencing therapeutic outcomes and associated risks.

**Table 4:** Stratification of frailty levels and immunotherapy response in elderly cancer patients.

Frailty level	Age range	G8 score	Key immunological features	Representative biomarkers	Degree of immunosuppression	Expected treatment tolerance	Ref.
Mild Frailty	65–74 years	15–17	Mild thymic atrophy; relative preservation of naïve T cells; higher proportion of CD28^+^ T cells	IL-6 < 5 pg/mL; CRP < 3 mg/L; slight increase in MDSCs; Tregs baseline +10–20%	Mild	Response rate 25–30%	(48, 49)
Moderate Frailty	70–84 years or ≥3 comorbidities	11–14	Moderate thymic atrophy; reduced TCR diversity; increased CD57^+^ T cells	IL-6 5–15 pg/mL; CRP 3–10 mg/L; MDSCs increased 1.5–2×; Tregs baseline +30–50%	Moderate	Response rate 15–25%	(43, 50)
Severe Frailty	≥85 years or G8 ≤10	≤10	Severe thymic atrophy; accumulation of terminally differentiated T cells; marked elevation of CD28^-^CD57^+^ T cells	IL-6 > 15 pg/mL; CRP > 10 mg/L; MDSCs >2× increase; Tregs baseline +50–80%	Severe	Response rate <15%	(34, 51)

### 1.2 Novel immunological challenges of oncolytic virus therapy in elderly cancer patients

**Figure 2:**
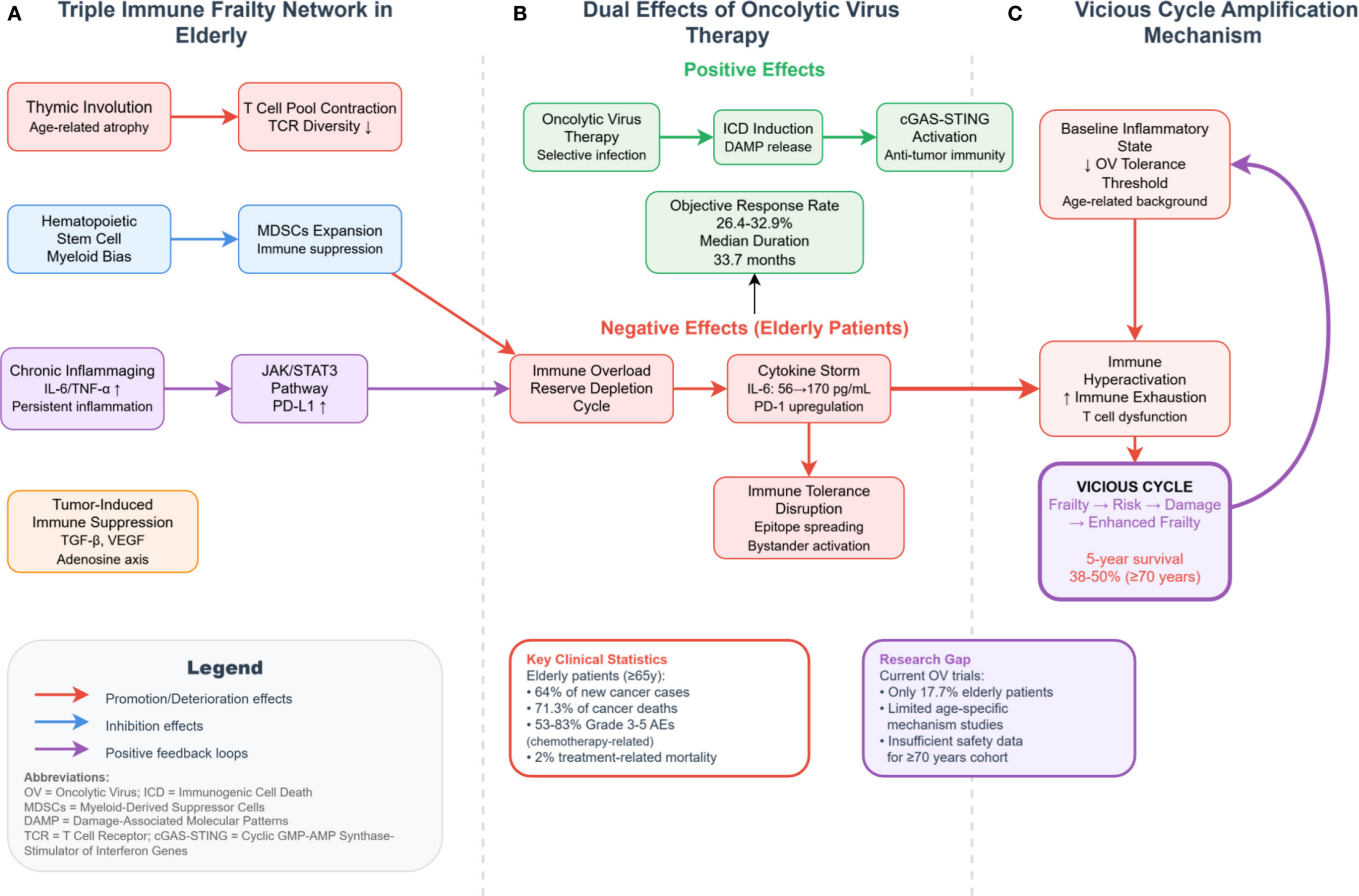
Molecular network of immune fragility and OV therapy interactions in elderly cancer patients. ICD, immunogenic cell death; DAMP, damage-associated molecular patterns; MDSCs, myeloid-derived suppressor cells; SASP, senescence-associated secretory phenotype; tEVs, tumor-derived extracellular vesicles; AICD, activation-induced cell death. This figure delineates the mechanistic interplay between immune fragility and OV therapy-associated risks in elderly cancer patients. **(A)** Triple-network of age-related immune dysfunction: Thymic involution reduces T-cell repertoire diversity; hematopoietic stem cell myeloid bias expands MDSCs; and chronic inflammaging (elevated IL-6/TNF-α) activates the JAK/STAT3–PD-L1 axis, which cooperates with tumor-derived immunosuppressive signals (e.g., TGF-β, VEGF, and adenosine pathway) to establish a coexisting immunosuppressive and inflammatory state. **(B)** Dual-edged effects of OV therapy: On the one hand, OVs trigger antitumor immunity via ICD–DAMP–cGAS–STING activation, facilitating immunologic conversion of cold tumors. On the other hand, their adverse effects are amplified in elderly patients, leading to: Immune overactivation and exhaustion cycles (e.g., surge in pro-inflammatory cytokines → PD-1 upregulation → T-cell dysfunction); Cytokine storms, with IL-6 levels rising from 56 to 170 pg/mL; Breakdown of immune tolerance via epitope spreading and bystander activation. **(C)** Feedback amplification loop: Preexisting low inflammatory thresholds in elderly patients reduce OV tolerability, while OV-induced immune hyperactivation further exacerbates immune exhaustion. This reinforces a vicious cycle of “fragility–risk–damage–increased fragility,” ultimately contributing to a 5-year survival rate of only 38–50% in patients aged 70 and above. Red arrows denote aggravating effects; blue arrows represent inhibitory effects; dashed arrows indicate positive feedback loops.

Oncolytic virus (OV) therapy presents several immunological challenges in elderly cancer patients, including immune overload, exhaustion of immune reserves, cytokine storm-driven inflammatory cascades, and disruption of self-tolerance. These adverse effects can severely compromise the already fragile immune landscape in aged individuals (**Figure 2**).

A major concern is the vicious cycle between immune overload and immune reserve exhaustion, which are tightly interconnected and dialectically unified (**Table 5**). Specifically, OV therapy triggers acute surges in pro-inflammatory cytokines such as IL-6, type I/II interferons, and TNF, which markedly upregulate Pdcd1 (PD-1) and Cd274 (PD-L1) transcription in T cells, leading to functional overload and subsequent depletion of immune reserves (18, 52). Moreover, combinatorial regimens involving T-VEC and chemotherapeutics (e.g., doxorubicin) can further escalate IL-6 levels from ~56 pg/mL to ~170 pg/mL, intensifying this immunological strain (53).

**Table 5:** The dual malignant cycle of immune overload and immunological resource exhaustion in elderly cancer patients under OV therapy.

Mechanism	Specific details	Study design	Data	Impact	Ref.
Surge of Acute Inflammatory Cytokines (CRS-like/“Localized Storm”)	Rapid elevation of IL-6, TNF-α, IFN-α/β amplifies innate immunity	i.p. oHSV-2 (OH2) in murine malignant ascites model	Significant IL-6 surge on days 4–9 post-treatment; IL-6R blockade reduces efficacy but reveals intense inflammation; scRNA-seq: Pdcd1/Cd274 upregulated	Inflammaging and impaired homeostasis increase risk of systemic collapse in elderly	(18)
Systemic Cytokine Cascade	Systemic elevation of IFN-α/β, IFN-γ, TNF-α	Phase I trial of IV pelareorep (reovirus) in solid tumors	IFNs and TNF increase post-treatment, indicating systemic immune cascade activation	Elderly patients with impaired type I IFN regulation and lowered inflammation threshold face higher exhaustion/adverse event risks	(52, 126)
High Antiviral Load → Antigen/Pathway Competition	Strong antiviral T cell/ISG responses upregulate PD-1/PD-L1, diverting resources	scRNA-seq of oHSV-2–treated ascites	Pdcd1/Cd274 transcription upregulated post-treatment, indicating exhaustion drift under intense antiviral pressure	Aged individuals with reduced naïve T pool and antiviral memory skew may exhibit resource hijacking	(18)
Metabolic Resource Exhaustion in Tumor-infiltrating T Cells	Glucose/mitochondrial depletion, lipid imbalance → impaired effector function	Vaccinia virus in melanoma-bearing mice	TILs show severe metabolic deficits post-VV treatment; leptin-loaded VV restores metabolism and tumor clearance	Aged T cells show reduced mitochondrial function and metabolic plasticity, increasing energy exhaustion risk	(56)
Macrophage/Myeloid Overload and Lipid Congestion	Tumor debris and cholesterol overload impair phagocytosis and TAM function	Glioma model; oncolytic adenovirus	OV-induced debris triggers TAM cholesterol overload; ApoA1-armed OV rescues phagocytosis and enhances tumor control	Lipid dysregulation and chronic inflammation in elderly promote myeloid congestion	(67)
Combination Therapy Exacerbates Inflammatory Load	Chemo or immune activators with OV further increase IL-6	In vitro: T-VEC/lysate + doxorubicin on SK29MEL melanoma cells	IL-6 increases from 56 to ~170 pg/mL; highest in combined chemo + T-VEC lysate	Multimorbidity and diminished hepatic/renal/marrow reserves make elderly more vulnerable to systemic inflammation	(53)
Elevated Baseline Inflammaging → Lower Exhaustion Threshold	Baseline IL-6/TNF-α high; naïve T cell decline; PD-1/TIM-3 ↑	Systematic reviews on immunosenescence in elderly & cancer	High IL-6 correlates with comorbidities/mortality; exhaustion/senescence markers increased	“Pre-sensitized” elderly immune background more prone to overload and exhaustion upon OV activation	(58)

Elderly individuals inherently exhibit elevated baseline levels of IL-6 and TNF-α, reduced naïve T-cell pools, and increased expression of exhaustion markers (e.g., PD-1, TIM-3), indicative of immunosenescence (54, 55). This aging-associated immune state exacerbates the risk of immune overload and exhaustion under OV therapy, contributing to lymphopenia and impaired T-cell metabolism (56–58). Additionally, OV-induced hematopoietic stem cell depletion worsens metabolic dysfunction and deepens immunosuppression (19).

Conversely, immune exhaustion can also precipitate further immune overload. OV treatment activates robust antiviral T-cell responses and upregulates interferon-stimulated genes (ISGs), fostering a suppressive immune milieu via checkpoint molecule induction. In elderly patients, prior viral exposures contribute to memory T-cell bias, reducing the naïve T-cell repertoire (59–61). OV-induced inflammation disproportionately burdens the remaining unskewed T cells, leading to their numerical expansion but diminished function (62–64). This paradoxical expansion is frequently followed by contraction via activation-induced cell death (AICD) (65, 66), culminating in accelerated immune reserve depletion. Moreover, tumor lysis by OVs generates debris accumulation, triggering cholesterol overload in tumor-associated macrophages and impairing their phagocytic capacity, further taxing immune resources (67).

OV therapy can also induce cytokine release syndrome (CRS), characterized by explosive surges of IL-6 and TNF-α within a short period (18). CRS represents one of the most life-threatening acute toxicities of OV therapy (68, 69). Despite prophylactic use of potent corticosteroids (e.g., dexamethasone) (70) or localized administration strategies (25), CRS manifestations such as fever, elevated AST, thrombocytopenia, and treatment interruptions remain common (20). The self-amplifying “inflammation–immunosuppression cycle” in the elderly, characterized by increased levels of immunosuppressive metabolites such as lactate, further amplifies the severity and long-term consequences of CRS (71–73). However, systematic age-specific incidence data remain lacking, limiting the ability to quantitatively assess the severity and exact frequency of adverse events in elderly patients (**Table 6**).

**Table 6:** Inflammatory cascade reactions (CRS/SIRS) induced by oncolytic viruses in elderly cancer patients.

Mechanism	Specific details	Evidence	Adverse events	Implications	Ref.
Systemic cytokine storm / CRS (Reovirus combination therapy)	Reovirus activates RIG-I/MDA5 → NF-κB/IRF3 signaling. Combination with proteasome inhibitors or immunotherapeutic agents amplifies T cell activation and proinflammatory cytokine release.	Clinical study in multiple myeloma (MM): Pelareorep + carfilzomib/dexamethasone. A review reported the first OV-associated cytokine storm in hematologic malignancies.	CRS reported in NCT02101944 (severe symptoms), along with fever and thrombocytopenia leading to treatment discontinuation. First documented OV-induced CRS in hematologic cancer.	Elderly MM patients often exhibit inflammaging and elevated baseline IL-6, potentially heightening CRS risk. CRP/IL-6 should be closely monitored and early intervention is essential.	(127)
Systemic CRS (VSV-modified OV)	VSV triggers RIG-I/MAVS and type I interferon signaling; IFN-β transgene further amplifies innate immune cascades.	Phase I trial in uveal melanoma (intratumoral + intravenous): CRS observed in one patient at the highest dose level.	Among 12 patients, CRS was observed in dose level 4 (DL4), accompanied by AST elevation and thrombocytopenia.	Elderly melanoma is common. IV administration or high-dose regimens pose greater risks. Close monitoring of vital signs and cytokines, with tiered dose escalation and early symptom management (e.g., hydration, antipyretics, tocilizumab/steroids if needed), is recommended.	(70)
Regional (intracavitary) cytokine storm with potential progression to systemic inflammation	HSV-2–based OV (OH2, GM-CSF arm) significantly upregulates IL-6, TNF, and IFN-γ. IL-6 identified as a key driver.	Murine peritoneal tumor model: OH2 administered intraperitoneally for malignant ascites.	IL-6 increased >4-fold by day 4 post-treatment, with enhanced CD8^+^/CD4^+^ infiltration. Early IL-6 blockade via tocilizumab reduced antitumor immunity.	Malignant ascites and serosal metastases are common in elderly patients with poor physiological reserve. Local OV therapy may induce regional cytokine storms that spill over systemically. Pre-emptive IL-6 blockade and fever management are advised; comorbidities should be carefully evaluated.	(18)
Transgenic cytokines (e.g., GM-CSF) and innate immune amplification → granulocyte activation and inflammation	Poxvirus-based OV (JX-594 / pexastimogene devacirepvec) encodes GM-CSF. DNA sensing via cGAS–STING → TBK1 → IRF3/NF-κB induces multiple cytokines.	Dose-escalation trials in liver cancer and others reported granulocytosis and flu-like symptoms, supporting systemic cytokine induction.	Dose-dependent granulocyte elevation and induction of IL-6/IFN-γ; systemic inflammatory symptoms commonly observed.	Elderly patients with baseline inflammation and reduced marrow reserves may be more susceptible to GM-CSF–induced neutrophil fluctuations and fever. Infection risk and concurrent use of agents like G-CSF should be carefully assessed.	(20, 126, 128)
Real-world pharmacovigilance signals: SIRS/infectious complications	HSV-1 (T-VEC) is primarily administered intralesionally; systemic symptoms such as fever and chills are common, but post-marketing data indicate risks of sepsis and encephalitis.	FAERS review (Q1 2004–Q3 2023, N=1138 reports).	Common signals: fever, flu-like symptoms, chills. Unexpected signals: sepsis, encephalitis, syncope, lymphadenopathy.	Elderly patients exhibit immune frailty and atypical infection presentations. Even local administration warrants rigorous evaluation for SIRS and infection (monitor temperature, HR, RR, WBC, CRP, procalcitonin).	(25)

Furthermore, OV therapy disrupts immune tolerance in elderly individuals. Rapid lysis of tumor cells releases large quantities of shared self-tumor antigens, which are cross-presented by antigen-presenting cells (APCs), eliciting secondary T/B cell responses against non-target self-epitopes and triggering autoimmune reactions (23, 74). Potent activation of dendritic cells and the upregulation of type I IFN and TLR signaling lower the threshold for immune tolerance, promoting bystander activation of autoreactive lymphocytes (22). Additionally, molecular mimicry between OV components and self-antigens drives T cell–mediated autoimmune cross-reactivity (75).

Genetically engineered OVs expressing immune modulators such as anti–CTLA-4 further amplify T-cell responses, intensifying autoimmune pathology and impairing immune tolerance (21). As a result, OV therapy has been associated with various immune-related adverse events (irAEs), including vitiligo, lupus vasculitis, psoriasis, pneumonitis, and encephalitis (25). These risks are significantly heightened when OVs are combined with immune checkpoint inhibitors, leading to increased rates of grade ≥3 treatment-related adverse events (24), and underscoring the compounded vulnerability of immune tolerance in aged hosts (76, 77).

**Table 7** provides a comprehensive overview of the mechanisms, evidence, and clinical data supporting OV-induced disruption of immune self-tolerance.

**Table 7:** Disruption of immune tolerance by oncolytic virus (OV) therapy in elderly cancer patients.

Mechanism	Details	Study type	Evidence	Implications	Ref.
Epitope spreading leading to secondary immune responses against self-antigens	Tumor lysis → exposure of tumor/self-shared antigens → cross-presentation by DCs → expansion of T/B cell responses	Classic review (shared mechanisms in autoimmunity and cancer)	Chronic inflammation and antigen release promote responses to "off-target" self-epitopes, explaining post-treatment autoimmune phenotypes (e.g., vitiligo)	Elderly patients exhibit fragile immune homeostasis with impaired antigen clearance and regulatory networks (e.g., Tregs, IL-10), increasing the risk of tolerance breach	(23)
Innate immune hyperactivation and bystander activation	OV/T-VEC encodes GM-CSF → DC activation; upregulation of type I IFNs and TLR signaling → passive activation of bystander T/B cells	Review in *Front Immunol* (OV-induced innate and adaptive responses)	Strong IFN and PRR activation lowers peripheral tolerance thresholds, triggering autoimmunity-related phenotypes	Inflammaging in elderly further lowers the immune activation threshold	(22)
Molecular mimicry and autoantibody generation	Structural/sequence similarity between viral and self-antigens → cross-reactivity	Review on viral infection and autoimmunity	Summarizes multiple pathways (molecular mimicry, epitope spreading, bystander activation) contributing to autoimmune responses	Thymic involution and reduced clonal deletion of autoreactive lymphocytes in elderly increase risk	(75)
Treg axis impairment and tolerance network disruption	OV induces costimulatory and inflammatory signals; combination with anti–CTLA-4/PD-1 further depletes Tregs	Studies using oHSV vector expressing/delivering anti–CTLA-4; combination with PD-1 blockade enhances systemic immunity	Tumor-targeted Treg-depleting strategies amplify T-cell effects but may lower the autoimmunity threshold	Age-related Treg dysfunction/redistribution makes elderly more prone to tolerance breakdown	(21, 129, 130)
Clinical phenotype: T-VEC–induced vitiligo (melanocyte autoimmunity)	Shared antigens between melanoma and melanocytes; epitope spreading and inflammatory microenvironment	Final OPTiM Phase III analysis (melanoma)	Immune-related AEs in 24/295 (8.1%); vitiligo in 18/295 (6.1%), mostly Grade 1–2; additional Grade 3 events (e.g., lupus vasculitis, psoriasis, pneumonitis)	High proportion of elderly in melanoma cohort highlights the presence of "manageable but real" autoimmune risk	(131)
Case series: T-VEC–induced vitiligo	Associated with durable responses	Case series (melanoma)	Reports of vitiligo-like lesions following injection, supporting the correlation between autoimmunity and tumor control	Fragile skin barriers in elderly necessitate dermatologic surveillance	(131)
Pharmacovigilance signals: endocrine and neuroimmune events	FAERS real-world analysis (2004 Q1–2023 Q3)	Detected safety signals for endocrine disorders; unexpected neuroinflammatory signals (e.g., encephalitis, ROR 11.8, 9 cases)	Higher reporting frequency in ≥60 age group (consistent with melanoma epidemiology) indicates need for enhanced monitoring	Beyond common endocrine irAEs, rare but serious neuroimmune events in elderly may lead to rapid functional decline or fatality; proactive risk assessment and monitoring strategies are essential	(25)
Immune axis amplification via PD-1 combination therapy → further tolerance erosion	“Viral ignition + PD-1 blockade” amplifies systemic T-cell responses	Phase III trials (e.g., MASTERKEY-265: T-VEC + pembrolizumab)	Though negative for primary endpoint, ≥Grade 3 treatment-related AEs occurred in ~20%, consistent with ICI AE profiles; autoimmune events include endocrine, dermatologic, pulmonary manifestations	Given elderly patients' comorbidities, vigilant monitoring of thyroid, liver, and pulmonary function is essential	(24)
Other OV platforms with tolerance-disruptive potential	VACV, Reovirus elicit strong IFN/inflammatory responses and are often used with ICIs	2024 review on VACV/combination strategies	Mechanistic and combinatorial data suggest a general risk of “overactivation → tolerance breach” (though mostly manageable)	For elderly, a low-intensity initiation with stepwise escalation and tight monitoring is recommended	(77, 132)

## 2 Discussion

Over the next 5–10 years, the immunological frailty of elderly cancer patients is expected to be progressively overcome (**Table 8**). Tumor-induced immunosuppression may be alleviated through a range of combination strategies, such as the use of the A2A receptor antagonist ciforadenant in conjunction with anti–PD-L1 therapy (78), CD73 inhibitors paired with immune checkpoint blockade (79, 80), and VEGF-Trap agents that correct dendritic cell differentiation defects (81). Furthermore, the development of novel drugs is anticipated based on evidence that the PDE5 inhibitor tadalafil effectively reduces MDSC and Treg levels (82).

**Table 8:** Strategies to address the three core dimensions of immune fragility in elderly cancer patients.

Core issue	Mechanism	Strategy	Evidence	Ref.
Tumor-induced immunosuppression	Adenosine pathway (CD39/73 → adenosine → A2A-mediated T cell inhibition)	A2A receptor antagonist (ciforadenant) ± anti–PD-L1	First-in-human studies show safety and potential benefit as monotherapy and in combination with PD-L1 inhibitors	(78)
		CD73 inhibitors (small molecules/antibodies), typically combined with PD-1/PD-L1 or A2A blockade	Comprehensive preclinical/clinical evidence supports their role in reversing adenosine-mediated suppression	(79, 80)
	VEGF impairs DC differentiation/function	Anti–VEGF/VEGFR or "vascular normalization" strategies	VEGF-Trap can correct DC differentiation but insufficient alone; optimal when combined with immunotherapy	(81)
	MDSC-mediated suppression	PDE5 inhibitors (e.g., tadalafil)	Reduced MDSC/Treg levels and enhanced tumor immunity in HNSCC patients	(82)
Age-related immunosenescence	Insufficient naive/memory T cell reserves	Recombinant human IL-7	Expands naive and central memory T cells; validated in HIV immunorestoration with broad relevance	(83)
	Immune metabolic/exhaustion pathways	Low-dose mTOR inhibitors (everolimus/rapamycin)	Enhanced flu vaccine response and reduced PD-1 expression in elderly volunteers	(84)
	Thymic involution	Reversible sex steroid ablation (SSA), thymic regeneration	Human/mouse studies show SSA promotes thymic output and immune reconstitution; reviewed in translational studies	(85, 133)
	Senescent T cell phenotype	Metformin	Human trials show reversal of T cell aging (quantity/function/telomerase/transcriptome)	(85)
Synergistic vicious cycle: IL-6/STAT3 upregulation, pro-inflammatory yet suppressive	Cytokine release syndrome (CRS) management	Tocilizumab (IL-6R blockade) ± corticosteroids	First-line treatment validated in CAR-T/ICI-associated CRS	(68, 69)
	Refractory or steroid-resistant CRS	JAK inhibitors (e.g., ruxolitinib)	Clinical and retrospective studies (small cohorts) show rapid cytokine reduction and CRS control	(86–90)
	Pharmacological control of overactivation	Stepwise dose escalation, local/intratumoral delivery	General risk-mitigation strategy to lower systemic inflammation peaks, best used in combination with the above agents	(69)

Age-related immunosenescence is likely to be addressed by augmenting the quantity and quality of immune cells. Quantitative improvement may be achieved using cytokines such as recombinant human IL-7 to expand naïve and central memory T cells (83). Qualitative enhancement may involve low-dose mTOR inhibitors to downregulate PD-1 expression in T cells (84) and pharmacological agents such as metformin to attenuate T cell senescence through multidimensional mechanisms (85). In addition, reversible sex steroid ablation (SSA) and thymic regeneration approaches are under exploration to restore immune competency (85), providing a multifaceted strategy to counter immunosenescence.

To interrupt the vicious cycle between immunosuppression and immune exhaustion, standard treatment regimens such as tocilizumab combined with glucocorticoids—commonly used for CRS induced by CAR-T or checkpoint inhibitors—will be expanded (68, 69). JAK inhibitors like ruxolitinib, which can rapidly suppress cytokine surges and alleviate CRS, are also being investigated for broader application in immune-related toxicity control (86–90), paving the way for the development of next-generation interventions.

In parallel, the three core immune-related challenges induced by oncolytic virus (OV) therapy in elderly cancer patients will likely be tackled within the coming decade (**Table 9**). To break the bidirectional vicious cycle of immune overload and immune reserve exhaustion, emerging strategies include pharmacokinetic-based dosing (“controlled-peak” regimens), intratumoral administration to enhance local immune activation and reduce systemic toxicity, and low-dose fractionated schedules that optimize efficacy–safety ratios (91–93). Additional approaches involve enhancing cholesterol efflux and metabolic reprogramming to activate the ApoA1/ABCA1 pathway and improve macrophage phagocytosis and macrophage–T cell synergy (67), as well as IL-7–mediated expansion of T cell pools and diversification of the TCR repertoire to “replenish immune reserves” (83, 94, 95).

**Table 9:** Strategies to address OV-related risks in elderly cancer patients.

Core issue	Mechanism	Specific strategies	Evidence	Ref.
Bidirectional vicious cycle of immune overactivation and reserve exhaustion	Systemic pro-inflammatory surges upregulate PD-1 and ISG expression, leading to T cell dysfunction; persistent antigen load or high dosing intensity drives chronic stimulation	Pharmacokinetic-controlled delivery: stepwise dose escalation, intratumoral/intracavitary administration, and low starting dose strategies	Reviews and clinical practice highlight that intratumoral OV administration enhances local immune activation while minimizing systemic toxicity. Several studies suggest that low-dose or fractionated regimens can offer better efficacy-safety ratios in specific OV/immunotherapy settings	(91–93)
	Macrophage cholesterol overload → impaired phagocytosis, amplified immune overactivation/exhaustion	Cholesterol efflux/metabolic remodeling (enhancement of ApoA1/ABCA1 axis; cyclodextrin-HDL nanotechnology, currently at translational stage)	Enhancing cholesterol efflux improves tumor control and restores macrophage–T cell cooperation. Nanoparticle and gene-engineering approaches have significantly improved phagocytic and effector function in preclinical models	(67)
	Reduced T cell repertoire and diversity in the elderly → increased susceptibility to exhaustion	IL-7–mediated immune reconstitution during recovery/maintenance phases	IL-7 expands naïve and memory T cells, broadens TCR diversity, and has been shown to enhance immune reserves in both human and disease models. It also shows compatibility with vaccines and cell-based therapies	(83, 94, 95)
Uncontrolled cytokine release syndrome (CRS)	IL-6–driven acute inflammatory amplification; corticosteroid-refractory in some cases	Tocilizumab (IL-6R blockade) as first-line; supportive care and graded management (per ASCO/SITC guidelines)	Multicenter and retrospective studies support rapid CRS resolution with IL-6R blockade, in line with guideline recommendations for grading and administration timing	(87, 89, 96, 97)
	IL-1/macrophage-driven corticosteroid-refractory CRS	Anakinra (IL-1R antagonist) for salvage/prevention	Clinical and translational studies show IL-1 blockade mitigates CRS and neurotoxicity, effective in cases unresponsive to steroids/tocilizumab	(86, 88)
	Multi-cytokine cascades, corticosteroid-refractory	Ruxolitinib (JAK1/2 inhibitor)	Small-scale prospective/retrospective studies suggest rapid cytokine reduction and CRS control without significantly impairing antitumor effects (timing and dosage require caution)	(87, 89, 90)
Breakdown of self-tolerance (epitope spreading, bystander activation, etc.)	Broad immune overactivation leading to organ-specific immune-related adverse events (irAEs)	Graded management: treatment interruption/dose reduction; corticosteroids as the mainstay; organ-specific second-line agents (e.g., infliximab for colitis, mycophenolate for hepatitis, IVIG/rituximab for cutaneous, neurological, or hematologic toxicities)	Authoritative irAE management guidelines for ICIs provide organ-specific protocols, applicable to irAEs induced by OV combination/sequential therapies	(8, 9)
	Myocarditis/severe organ toxicity refractory to corticosteroids	Abatacept (CTLA-4-Ig) ± ruxolitinib	Case reports and dose-finding studies suggest abatacept reverses ICI-induced myocarditis. Ongoing trials aim to optimize dosing; potential salvage option for steroid-refractory severe irAEs	(98–100)
	Systemic bystander activation/epitope spreading risk	Route optimization: prioritize intratumoral injection; use regional delivery when necessary to reduce systemic exposure	Intratumoral OV/immunotherapy delivery has shown robust immune activation and lower systemic toxicity across multiple tumor types	(101, 102)

The challenge of cytokine storm (CRS) will be addressed through first-line use of IL-6R blockers such as tocilizumab for rapid symptom relief (87, 89, 96, 97). For steroid-refractory CRS, IL-1R blockers (e.g., anakinra) and JAK1/2 inhibitors (e.g., ruxolitinib) will be employed to mitigate cytokine levels while preserving antitumor activity (86–90). This comprehensive, multi-tiered control framework is essential for CRS prevention and management.

The disruption of immune tolerance associated with OV-based regimens will be managed using a structured irAE management protocol based on current ICI guidelines. First-line glucocorticoids will serve as foundational therapy, with second-line, organ-specific agents such as infliximab for colitis, mycophenolate mofetil for hepatitis, and IVIG/rituximab for dermatologic and hematologic toxicity (8, 9). For cardiotoxicity, the combination of abatacept (CTLA-4-Ig) and ruxolitinib has shown promise in reversing ICI-induced myocarditis (98–100). Drug delivery route optimization—via intratumoral or regional administration when possible—will further reduce systemic exposure and minimize the risk of bystander immune activation (101, 102), offering a holistic strategy to restore immune tolerance.

Despite these advancements, the unique immune landscape of elderly cancer patients remains marginalized (**Table 10**). Due to comorbidities, suboptimal biomarker profiles, and compromised performance status (103), elderly individuals comprise only 17.7% of early-phase clinical trial participants (14). Within the context of OV therapy, elderly patients account for 34.4% of immune-related adverse events (25), and are at heightened risk of severe complications—such as disseminated HSV infection or encephalitis—even with localized treatments like T-VEC (26).

**Table 10:** Overlooked issues of immunological vulnerability in elderly cancer patients.

Evidence	Details	Implications	Recommendations	Ref.
EGALICAN-2 study in France: patients aged ≥70 accounted for only 17.7% of early-phase clinical trials	Systematic underrepresentation of older adults results in limited data on immunotoxicity and efficacy	Extrapolation from younger cohorts may lead to overtreatment, undertreatment, or insufficient monitoring	Eliminate unnecessary upper age limits; define elderly-specific endpoints (e.g., functional status, quality of life, treatment tolerance); establish dedicated older adult cohorts	(14)
Community-based research: “ineligibility” for clinical trials is more common in elderly patients (due to comorbidities, unmet biomarkers, poor performance status)	Inclusion/exclusion criteria neglect typical physiological and immunological features of aging (e.g., chronic inflammation, comorbidities, biopsy difficulties)	Further exacerbates evidence gaps and uncertainty in therapeutic decision-making	Loosen laboratory and biomarker thresholds; adopt pragmatic trial designs and decentralized biospecimen collection	(103)
Early fatal signals of ICI-related myocarditis (case reports and pharmacovigilance data)	Cardiovascular aging and immune senescence increase baseline risk, complicating early detection	Sudden death and treatment discontinuation	Routine baseline and early-cycle screening with hs-troponin, ECG, and echocardiography; joint oncology–cardiology clinics; intensive monitoring and rapid immunosuppression escalation for high-risk populations	(108–110)
Geriatric assessment (GA) has been validated in RCTs to reduce severe toxicity and improve outcomes, but remains underutilized in practice	Differences in immune/inflammatory reserve are rarely assessed or managed	Higher rates of adverse events, hospitalization, and poor treatment adherence	Incorporate GA as standard practice before systemic therapy in all patients aged ≥65; use results to guide dosing, regimens, and supportive care strategies	(6, 104–107)
Real-world pharmacovigilance of T-VEC: beyond fever, reports include sepsis, encephalitis, syncope, and lymphadenopathy; 34.4% of cases involved older adults	Decline in immune tolerance and risk of latent virus reactivation are underestimated in the elderly	Severe infectious complications, treatment discontinuation, or death	Perform virological/immunological stratification before OV therapy; administer first dose under inpatient monitoring in elderly or immunocompromised patients; develop robust antiviral contingency plans	(25)
Case reports of disseminated HSV infection and encephalitis after T-VEC	Even local administration can trigger systemic infection, with heightened risk in elderly or immunocompromised individuals	Severe infections and neurological complications	Screen for prior herpes zoster/HSV infections and immunosuppressive conditions; consider prophylactic antivirals where appropriate	(26)

The following notations and conventions are used throughout the tables in this manuscript. Arrows and symbols represent biological or functional relationships: ↑ indicates upregulation or increased levels; ↓ denotes downregulation or decreased levels; → signifies causality, functional directionality, or pathway flow; ↔ reflects bidirectional interactions or feedback loops; × denotes interaction, additive effects, or joint causation; ± implies optional presence or monotherapy versus combination use (e.g., Drug A ± Drug B); + indicates combination or co-administration (e.g., A + B); ≥ and ≤ represent threshold boundaries; ≈ or ~ indicate approximate values; “/” denotes alternatives or parallel items depending on context; “–” (en dash) is used for ranges or compound terms (not a negative sign); NA or — signifies data unavailability or non-applicability.

Statistical and study design indicators include: *n* (sample size), *P* (p-value, two-sided unless otherwise stated), *HR* (hazard ratio; >1 indicates increased risk or adverse outcome), *95% CI* (95% confidence interval), *ROR* (reporting odds ratio, used for signal strength in pharmacovigilance), and *RCT* (randomized controlled trial). Drug administration routes are abbreviated as *i.v.* (intravenous), *i.p.* (intraperitoneal), and *i.t.* (intratumoral). "5-year survival" denotes the 5-year survival rate. All numerical values and significance are reported as per the original studies.

Regarding populations and experimental settings, “human” or “patient” refers to clinical data, while “mouse” indicates animal models. “In vitro” denotes cell-based assays; “intracavitary” refers to administration into body cavities (e.g., peritoneal, pleural); “ascites” refers to malignant ascites. TME indicates the tumor microenvironment, and TIL denotes tumor-infiltrating lymphocytes. If multiple species or contexts are involved in a single entry, specific details are provided under the relevant columns such as "Evidence," "Study Design," or "Subjects."

Adverse event terms such as *CRS* (cytokine release syndrome), *SIRS* (systemic inflammatory response syndrome), and *irAEs* (immune-related adverse events) are used in accordance with immunotherapy-related safety classifications. Safety signals arising from drug platforms, viral vectors, or combination regimens are interpreted based on the severity grading and recommended management per the cited studies or clinical guidelines.

Gene and protein names follow the original source nomenclature (e.g., HGNC, UniProt). Terms like "upregulated," "downregulated," "associated," or "correlated" summarize statistically significant or mechanistically supported findings. Where entries are inferential or based on integrated mechanistic hypotheses, the evidence level and scope of applicability are detailed in the respective “Evidence” or “Implications” columns.

However, geriatric assessment (GA), a tool proven to reduce severe toxicity and improve outcomes, remains underutilized in routine clinical practice. This oversight leads to insufficient immunological risk assessment, ultimately contributing to elevated rates of adverse events, hospitalizations, treatment non-compliance (6, 104–107), and even unexpected mortality or treatment discontinuation (108–110).

Therefore, at the clinical level, we recommend establishing a three-tiered frailty-based dosing strategy: standard-dose regimens for mildly frail patients, 25–50% dose reduction for moderately frail patients, and cautious risk–benefit evaluation for severely frail patients. In addition, a CRS early-warning system should be implemented, focusing on three critical markers: IL-6 levels, body temperature fluctuations, and platelet count. For high-risk individuals with a G8 score ≤14, enhanced monitoring protocols should be applied. Moreover, geriatric assessment (GA) should be standardized as a prerequisite for OV treatment in patients aged ≥65 years. In terms of clinical research, elderly patients should comprise ≥30% of enrolled participants, with at least three ongoing trials dedicated to dose optimization in this population. Furthermore, a dedicated OV safety database containing ≥200 elderly cases should be established to quantify age-specific risks and support precision medicine initiatives.

## Data Availability

The original contributions presented in the study are included in the article/supplementary material. Further inquiries can be directed to the corresponding author.
